# Fast machine-learning online optimization of ultra-cold-atom experiments

**DOI:** 10.1038/srep25890

**Published:** 2016-05-16

**Authors:** P. B. Wigley, P. J. Everitt, A. van den Hengel, J. W. Bastian, M. A. Sooriyabandara, G. D. McDonald, K. S. Hardman, C. D. Quinlivan, P. Manju, C. C. N. Kuhn, I. R. Petersen, A. N. Luiten, J. J. Hope, N. P. Robins, M. R. Hush

**Affiliations:** 1 Quantum Sensors and Atomlaser Lab, Department of Quantum Science, Research School of Physics and Engineering, The Australian National University, Acton, 2601, Australia; 2 Australian Centre for Visual Technologies, University of Adelaide, Adelaide, 5005, Australia; 3 School of Computer Science, University of Adelaide, Adelaide, 5005, Australia; 4 School of Engineering and Information Technology, University of New South Wales at the Australian Defence Force Academy, Canberra, 2600, Australia; 5 Institute for Photonics & Advanced Sensing, School of Physical Sciences,The University of Adelaide, Adelaide, 5005, Australia; 6 Department of Quantum Science, Australian National University, Canberra, 2601, Australia

## Abstract

We apply an online optimization process based on machine learning to the production of Bose-Einstein condensates (BEC). BEC is typically created with an exponential evaporation ramp that is optimal for ergodic dynamics with two-body s-wave interactions and no other loss rates, but likely sub-optimal for real experiments. Through repeated machine-controlled scientific experimentation and observations our ‘learner’ discovers an optimal evaporation ramp for BEC production. In contrast to previous work, our learner uses a Gaussian process to develop a statistical model of the relationship between the parameters it controls and the quality of the BEC produced. We demonstrate that the Gaussian process machine learner is able to discover a ramp that produces high quality BECs in 10 times fewer iterations than a previously used online optimization technique. Furthermore, we show the internal model developed can be used to determine which parameters are essential in BEC creation and which are unimportant, providing insight into the optimization process of the system.

Experimental research into quantum phenomena often requires the optimization of resources or processes in the face of complex underlying dynamics and shifting environments. For example, creating large Bose-Einstein condensates (BECs) with short duty cycles is one of the keys to improving the sensitivity of cold-atom based sensors^1^ or for performing scientific investigation into condensed matter phases^2^, many-body physics^3^ and non-equilibrium dynamics^4^. The standard process of BEC production is evaporative cooling^5^. Microscopic semi-classical theory exists to describe this process^6^, but it can oversimplify the dynamics and miss more complex and effective methods of performing evaporation. For example, Shobu *et al.*^7^ found circumventing higher order inelastic collisions can produce large condensates. ‘Tricks’ like this are likely to exist for other species with complicated scattering processes^8^, but discovery is only possible by experimentation. We automate this process of discovery with *machine-learning* online optimization (MLOO). What distinguishes our approach from previous methods for optimization is that we seek to develop a statistical model of the relationship between parameters and the outcome of the experiment. We demonstrate that MLOO can discover condensation with less experiments than a competing optimization method and provide insight into which parameters are important in achieving condensation.

Online optimization (OO), with mostly genetic^9^,^10^,^11^,^12^,^13^,^14^,^15^,^16^,^17^,^18^,^19^,^20^,^21^,^22^ but also gradient^23^ and hybrid solvers^24^,^25^, has been used to enhance a variety of quantum experiments. Here we use online to mean optimization that is performed in real time with the experiment. What distinguishes our approach is that it does not only seek to optimize the experiment, but also creates an internal model that is able to predict the performance of future experiments given any set of parameters. This is achieved by modeling the experiment using a Gaussian process (GP)^26^. Our algorithm both fits the model to previous observations, and chooses to do future experiments that will best refine its model, making it an automation of scientific method. Online machine learning (OML) with GPs^26^,^27^,^28^,^29^,^30^ has been applied in a variety of areas including robotics^31^,^32^, vision^33^, industrial chemistry^34^,^35^ and biochemistry^36^. However, in all of these cases, the focus was not on optimization. Rather, the goal was the development of an accurate model. We combine the advantages of OML with the motivation of OO. The resultant MLOO algorithm has the following advantages: every experimental observation is used to improve the GP model, and uncertainties in the measurements are correctly accounted for; our algorithm will find global minima, but exploration is not random, new parameters are picked with knowledge of where the learner is most uncertain; the learner provides a visualization of the resource’s quality as function of the parameters that can inform experimentalists on how to best develop future optimization experiments.

## Methods

### Experiment

**Figure 1:**
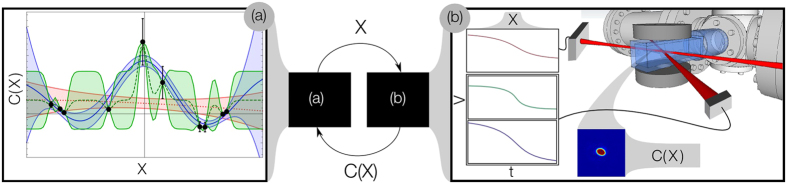
The experiment and the ‘learner’ form a closed loop. The learner produces a parameter set, *X*, for the experiment to test, these are converted into cooling ramps and used to perform an experiment. After the evaporation process is finished, an image of the cold atoms taken is used to calculate a cost function based on its quality as a resource *C*(*X*). *C*(*X*) is then fed back to the learner. The learner uses a GP to model the relationship between the input parameter values and the cost function values produced by the experiment. This model depends on a set of correlation lengths, or hyperparameters. Part (**a**) of the figure plots a set of observed costs (black circles with bars for uncertainty) with three possible GP models fit to the data: one with a long correlation length (red dotted), a medium correlation (blue solid) and a short correlation length (green dashed). Each GP is illustrated by a mean cost function bracketed by two curves indicating the function +/− one standard deviation from the mean. The correlation lengths affect both the mean and uncertainty of the model; note that the uncertainty approaches zero near the observed points. A final cost function is produced as a weighted average over the correlation lengths. This model is used to pick the next parameters *X* for the experiment.

The experimental apparatus is described in detail in^37^. Initially ^87^Rb atoms are cooled in a combined 2D and 3D MOT system and subsequently cooled further by RF (radio frequency) evaporation. The cloud is then loaded into a cross beam optical dipole trap for the final evaporation stage. It is this stage that is the subject of the optimization process. At this point, the sample contains 4 × 10^7^ atoms at a temperature of ~5 *μ*K with a phase space density of ~0.05. The cross dipole trap is formed from two intersecting 1090 nm and 1064 nm lasers with approximate waists of 350 *μ*m and 300 *μ*m respectively producing a trap with frequencies 185 × 185 × 40 Hz. The depth of the cross trap is determined by the intensity of the two beams and is found to be approximately 70 *μ*K. The 1064 nm beam is controlled by varying the current to the laser, while the 1090 nm beam is controlled using the current and a waveplate rotation stage combined with a polarizing beamsplitter to provide additional power attenuation while maintaining mode stability. A diagram of the experimental set up is shown in Fig. 1. Normally the power to these beams is ramped down over time, thereby lowering the walls of the trap and allowing the higher energy atoms to leak out. The remaining atoms rethermalize to a lower temperature, enabling cooling. Once the gas has been cooled to temperatures on the order of nK, a phase transition occurs, and a macroscopic number of atoms start to occupy the same quantum state. This transition is called Bose-Einstein condensation^38^. We hand over control of these ramps to the MLOO. We consider two parameterizations: one simple, where we only control the start and end points of a linear interpolation; and one complex, where we add variable quadratic, cubic and quartic corrections to the simple case (see Supplemental Equations).

### Performance Measure

**Figure 2:**
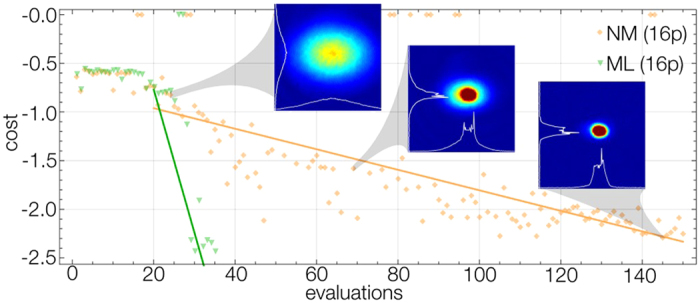
The optimization of the evaporation stage of creating a BEC using the complex 16 parameter scheme. The first 20 evaluations are an initial training run using a simple Nelder-Mead algorithm. The machine learning algorithm (green) then quickly optimizes to BEC. The insets show the different regimes experienced by the experiment, from a completely Gaussian thermal distribution, through the bimodal distribution containing a thermal background to the sharp edged BEC. The included cross-section illustrates how the cost decreases as the edges of the cloud get sharper.

The approach we propose is a form of supervised learning, meaning that we provide the learner with a number that quantifies the quality of the resource produced or in optimization terminology a cost that must be minimized. Naïvely one might try to use a measure based on temperature and particle number. However determining these quantities accurately near condensation is difficult when constrained to very few runs per parameter set. Instead, a technique was created to measure the width of the edges of the cloud. For thermal clouds this edge is broad, but as the sample cools and condenses these edges become sharper. To quantify this, an absorption image of the final state of the quantum gas is taken after a 30 ms expansion of the cloud, with the image providing the optical depth as a function of space. This absorption image is taken at resonance, resulting in saturation of the image (see Fig. 2). Whilst this makes determining peak density difficult, it ensures that the edges of the cloud are accurately determined. The cost is then calculated from all data between a lower and upper threshold optical depth. The lower threshold is determined by the noise in the system. The upper threshold is set slightly lower than the saturation level of the image. Only data from between the bounds is used and the cost is simply the average of these values. In practice this means the sharper the edges of the cloud, the lower the cost. Indeed, low quality thermal clouds have broad edges, whereas the ideal BEC has much sharper edges. Each parameter set is tested twice with the average of the two runs used for the cost. Tests of the variation in cost for a set of parameters run-to-run indicate they obey a Gaussian distribution. As such we are able to estimate the uncertainty from two runs as twice the range. In doing so, the chance we have underestimated the uncertainty will be 27%. We therefore also apply bounds to the uncertainty to eliminate outliers overly affecting the modeling process. The cost function can be evaluated as long as some atoms are present at the end of the evaporation run. In cases where the evaporation parameters produced no cloud twice for a set of parameters, we set the cost to a default high value.

### Algorithm

We treat the experiment as a stochastic process 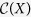 which is dependent on the parameters *X* = (*x*_1_,…, *x*_*M*_). When we make a measurement and determine a cost, we interpret this as a sample of this process *C*(*X*) with some associated uncertainty *U*(*X*). We define the set of all parameters, costs and uncertainty previously measured as 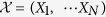, 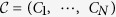 and 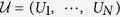 respectively and collectively refer to these sets as our observations 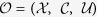. The aim of OO is to use previous observations 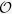 to plan future experiments in order to find a set of parameters that minimize the mean cost of the stochastic process 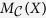. Unique to the MLOO approach, we first make an estimate of the stochastic process given our observations 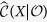, which is then used to determine what parameters to try next.

We model 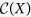 as a GP–a distribution over *functions*–with constant mean function and covariance defined by a squared exponential correlation function 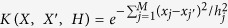 where *H* = (*h*_1_, …, *h*_*M*_) is a set of correlation lengths for each of the parameters. The mean function conditional on the observations 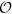 and correlation lengths *H* of our GP is: 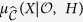, which is evaluated through a set of matrix operations^26^ (see Supplemental Equations). As we are using a GP, we can also get the variance of the functions conditioned on 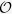 and *H*: 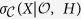
26. Both of these estimates depend on the correlation lengths *H*, normally referred to as the hyperparameters of our estimate. We assume that *H* is not known a priori and needs to be fitted online.

The correlation lengths *H* control the sensitivity of the model to each of the parameters, and relates to how much a parameter needs to be changed before it has a significant effect on the cost (see Fig. 1). A standard approach to fit *H* is maximum likelihood estimation^26^. Here, the hyperparameters are globally optimized over the likelihood of the parameters *H* given our observations 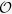, or 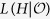
26 (see Supplemental Equations). However, when the data set is small there will often be multiple local optima for the hyperparameters whose likelihoods are comparable to the maximum. We term these hyperparameters the hypothesis set 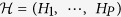 with corresponding likelihood set 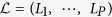.

To produce our final estimates for the mean function and variance we treat each hypothesis as a *particle*^30^, and perform a weighted average over 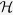. The weighted mean function is now defined as 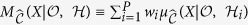 and weighted variance of the functions is 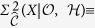 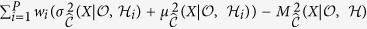, where 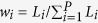 are the relative weights for the hyperparameters. Now that we have our final estimate for 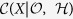, we need to determine an optimization strategy for picking the next set of parameters to test.

Consider the following two strategies: We could always test parameters that are predicted to minimize 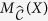, making our learner act as an ‘optimizer’. But this learner could get trapped in local minima and re-enforce its ignorance; Or we could test parameters that maximize 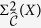 (i.e. where we are most uncertain), this would provide us with experimental data that helps us best refine our model and discriminate between the hypotheses, making our learner act like a ‘scientist’. But this learner may require a large number of trials to map the space and would not prioritize refinement of the global minima. We chose to implement a balanced strategy that repeatably sweeps between these two extremes by minimizing a biased cost function: 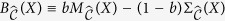, where the value for *b* is linearly increased from 0 to 1 in a cycle of length *Q*. During testing with synthetic data, we found sweeping the learner between acting like a ‘scientist’ (*b* = 0) and an ‘optimizer’ (*b* = 1) was more robust and efficient than fixing the learner to one strategy. When we minimized 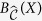 we also put bounds, set to 20% of the parameters maximum-minimum values, on the search relative to the last best measured *X*. We call these bounds a leash, as it restricts how fast the learner could change the parameters but did not stop it from exploring the full space (similar to trust-regions^39^,^40^). This was a technical requirement for our experiment: when a set of parameters was tested that was very different from the last set, the experiment almost always produced no atoms, meaning we had to assign a default cost that did not provide meaningful gradient information to the learner. Once the next set of parameters is determined they are sent to the experiment to be tested. After the resultant cost is measured this is then added to the observation set 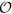 with *N* → *N* + 1 and the entire process is repeated.

As a benchmark for comparison, we also performed OO using a Nelder-Mead solver^41^, which has previously been used to optimize quantum gates^25^.

## Results

We demonstrate the performance of machine learning online optimization in comparison to the Nelder-Mead optimizer in Fig. 2. Here we used the complex parameterization for all 3 ramps, and added an extra parameter that controlled the total time of the ramps, resulting in 16 parameters. If we were to perform a brute force search and optimize the parameters to within a 10% accuracy of the parameters maximum-minimum bounds, the number of runs required would be 10^16^. The Nelder-Mead algorithm is able to find BEC much faster than this, in only 145 runs. The machine learning algorithm, on the other hand, is much faster. After the first 20 training runs, where the machine learning and Nelder-Mead algorithm use a common set of parameters, the machine learning algorithm converges in only 10 experiments.

The learner used in Fig. 2 only used the best hypothesis set when picking the next parameters, in other words we set *P* = 1. Evaluating multiple GPs is computationally expensive with so many parameters, so to save time we made this restriction. In spite of this, the learner discovered ramps that produced BEC in very few iterations. This is because the learner consistently fitted the correlation lengths of the 3 most important parameters–the end points of the ramps–very quickly. However, we found the other correlation lengths were not estimated well and would not converge, even after a BEC was found. This meant that we were unable to make useful predictions about the cost landscape and we could not reliably determine what parameters were least important. The final optimized parameters produced a condensate with 5 × 10^5^ atoms.

**Figure 3:**
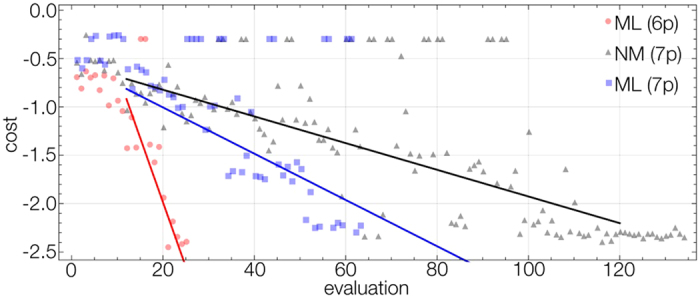
Optimization of evaporation curves to produce a BEC. The first 2N evaluations use a simple Nelder-Mead algorithm to learn about the cost space. The machine learning algorithm (red and blue) optimizes to BEC faster than the Nelder-Mead (black). By utilizing the machine learning model a parameter is eliminated and the convergence improves (red).

Gramacy *et al.*^30^ have suggested that making good online estimation of the GP correlation lengths requires multiple particles. We considered achieving this goal in a different experiment as shown in Fig. 3. Here we used a learner with many particles *P* = 16, but had to use the simple parameterization for the ramps to save computational time. This resulted in a total of 7 parameters. We can see again the overall trend for the machine learner is still faster than Nelder-Mead, but less pronounced. More carefully estimating the correlation lengths has hindered the convergence rate compared to the 16 parameter case. Nevertheless, as we now have a more reliable estimate of the correlation lengths we can take advantage of a different feature of the learner.

**Figure 4:**
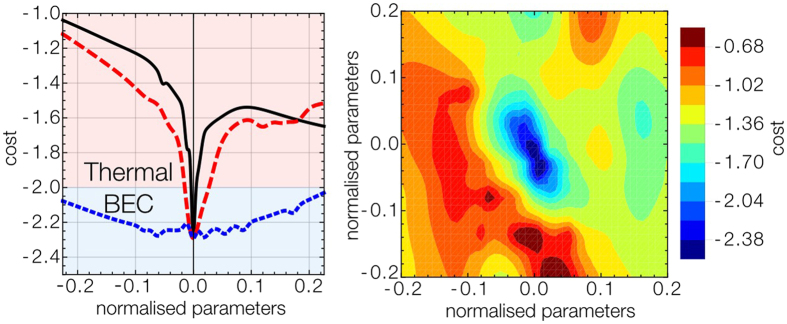
Plots of the cross sections through the minima of the cost landscape as predicted by the learner. In (**a**) the predicted cost is shown as a function of the end of the polarization ramp (red), the end of the dipole beam ramp (green) and the unconnected parameter (blue). The learner correctly identifies that the unconnected parameter does not have a significant effect on the production of BEC. In (**b**) a cross section of the 2 most sensitive parameters are plotted against cost.

In Fig. 4(a) we show estimates of the cost landscape as 1D cross sections about the best measured point. We plot the two most sensitive parameters and the least. We can see the least sensitive parameter appears to have no effect on the production of a BEC. This parameter corresponds to an intentionally added 7th parameter of the system that controls nothing in the experiment. Figure 4(a) shows the learner successfully identified this, even with such a small data set. After making this observation we can then reconsider the design of the optimization process and eliminate this parameter from the experiment.

In Fig. 3 we plot the machine learner optimization run with *P* = 16 but now with only 6 parameters. We can see the learner converges more rapidly than the 7 parameter case, and even produces a higher quality BEC. As the learner no longer takes extra runs to determine the importance of the useless 7^th^ parameter, it achieves BEC rapidly. We plot a 2D cross section of the landscape against the two most sensitive parameters in Fig. 4(b) generated from the 6 parameter machine learning run. We can see there is a very sharp transition to BEC, as it exists in a very deep valley of the landscape.

The optimum values for each parameter of the 16 parameter MLOO run are shown in a table in the supplementary material. Plots of the optimal ramps for each of the five optimization runs discussed are also shown and display cases where the optimum ramp is non-monotonically decreasing. The experimental controls adjust the shape of the trapping beams in a non-trivial manner, so this does not ensure that the trap depth experienced by the BEC was also non-monotonically decreasing. A key strength of the MLOO process is that we were able to find the ramps for the control that maximized the BEC without performing a lengthy characterization of relationship between the controls and the potential. Instead, the MLOO directly characterizes the relationship between the controls and the quality of the outcome. This significantly decreases the system identification and analysis overhead when optimizing an experiment.

The topology of optimization landscapes has been suggested as an explanation for the profound discrepancy between the number of experiments required to do a brute force search, and the number of experiments required in practice when using OO. Specifically, in laser-aided quantum chemistry, under the assumption of controllability it has been proven that landscapes are ‘trap-free’^42^ (there has been further refinement^43^,^44^,^45^,^46^,^47^ and debate^48^,^49^,^50^ on the generality of the result). In our experiment we observed Nelder-Mead always found condensation, albeit slower than MLOO, even though it is a local solver susceptible to being trapped. This suggests our landscape is also ‘trap-free’, and perhaps there is a universal principle for all quantum OO systems.

The MLOO algorithm we developed is available online^51^ (it uses^52^ to evaluate the GPs); it can be immediately applied to experiments that have previously used OO: quantum chemistry^9^, femtosecond physics^13^, and quantum computing^25^. Indeed, any automated experiment with a resource of measurable quality can be enhanced using MLOO.

## Additional Information

**How to cite this article**: Wigley, P. B. *et al.* Fast machine-learning online optimization of ultra-cold-atom experiments. *Sci. Rep.* **6**, 25890; doi: 10.1038/srep25890 (2016).

## Supplementary Material

Supplementary Information
