# Supplementary material for: Fast machine-learning online optimization of ultra-cold-atom experiments
Source: Sci Rep. 2016 May 16;6:25890. doi: 10.1038/srep25890 (PMC4867626; doi:10.1038/srep25890)
Supplement: Supplementary Information [file srep25890-s1.pdf]

Fast machine-learning online optimization of ultra-cold-atom experiments: Supplemental Material

P. B. Wigley[1], P. J. Everitt[1], A. van den Hengel[2], J. W. Bastian[3], M. A. Sooriyabandara[1], G. D. McDonald[1], K. S. Hardman[1], C. D. Quinlivan[1], P. Manju[1], C. C. N. Kuhn[1], I. R. Petersen[4], A. Luiten[5], J. J. Hope[6], N. P. Robins[1], and M. R. Hush[7,*]

[1]Quantum Sensors and Atomlaser Lab, Department of Quantum Science, Australian National University, Canberra, 2601, Australia
[2]Australian Centre for Robotic Vision, University of Adelaide, Adelaide, 5005, Australia
[3]School of Computer Science, University of Adelaide, Adelaide, 5005, Australia
[4]School of Engineering and Information Technology, University of New South Wales at the Australian Defence Force Academy, Canberra, 2600, Australia
[5]Institute for Photonics & Advanced Sensing, The School of Chemistry and Physics,The University of Adelaide, Adelaide, 5005, Australia
[6]Department of Quantum Science, Australian National University, Canberra, 2601, Australia
[7]School of Engineering and Information Technology, University of New South Wales at the Australian Defence Force Academy, Canberra, 2600, Australia
*M.Hush@adfa.edu.au

Gaussian process evaluation

In practice, evaluating a Gaussian process (GP) reduces to a set of matrix operations whose derivation is given by Rasmussen *et al.*[1] in section 2.7. Consider N previous experiments have been performed with parameter sets $\mathscr{X} = (X_1, \cdots X_N)$ (each $X_j = (x_{1,j}, \cdots x_{M,j})$), measured costs $\mathscr{C} = (C_1, \cdots, C_N)$ and uncertainties $\mathscr{U} = (U_1, \cdots, U_N)$. We refer to the set of this data as our observations $\mathscr{O} = (\mathscr{X}, \mathscr{C}, \mathscr{U})$. We fit a GP to these observations with constant function offset β and covariance defined by a squared exponential correlation function $K(X_p, X_q, H) = e^{-\sum_{j=1}^{M}(x_{j,p}-x_{j,q})^2/h_j^2}$ where $H = (h_1, \cdots, h_M)$ are the hyperparameters of the model.

The mean function and variance of the functions are:

$$\mu_{\hat{\mathscr{C}}}(X|\mathscr{O},H) = \beta + r(X)^T\gamma \tag{1}$$

$$\sigma^2_{\hat{\mathscr{C}}}(X|\mathscr{O},H) = \sigma^2_{\mathscr{C}}(1 - r(X)^T R^{-1} r(X) + (j^T R^{-1} j)^{-1}(j^T R^{-1} r(X) - 1)^2) \tag{2}$$

where $\sigma^2_{\mathscr{C}}$ is the variance of the costs $\mathscr{C}$, and we define the constant $\beta \equiv (j^T R^{-1} j)^{-1} j^T R^{-1} Y$, the $N \times 1$ vector $r(X)$ such that $\{r(X)\}_{1,i} = K(X, X_i, H)$, the $N \times 1$ vector $\gamma \equiv R^{-1}(Y - j\beta)$, the $N \times 1$ vector Y of the costs defined by $\{Y\}_{1,i} = C_i$, the $N \times 1$ vector $\{j\}_{1,i} = 1$, the $N \times N$ matrix R defined as $\{R\}_{i,j} = K(X_i, X_j, H) + \delta_{i,j} U_i^2$, and where $\delta_{i,j}$ is the Kronecker delta function. $\{\cdot\}_{i,j}$ is our notation for the ith row and jth column of a matrix or vector.

When finding the most likely hyperparameters we maximize the likelihood function. The likelihood $L(H|\mathscr{O})$ is defined as the probability of the costs given the parameters, uncertainties and hyperparameters: $P(\mathscr{C}|\mathscr{X}, \mathscr{U}, H)$, the log of which is:

$$\log P(\mathscr{C}|\mathscr{X},\mathscr{U},H) = \frac{1}{2}(-\log|R| - \log j^T R^{-1} j - (N-1)\log 2\pi - Y^T(R^{-1} - (j^T R^{-1} j)^{-1} R^{-1} j j^T R^{-1})Y) \tag{3}$$

Parameterizations of evaporation ramps

The simple parameterization of the evaporation ramps is

$$\mathscr{R}_s(y_i, y_f, t_f) = y_i + (y_f - y_i)\frac{t}{t_f} \tag{4}$$

where y_i and y_f specify the start and end amplitudes of the ramps and t_f specifies the length in time.

The complex parameterization an extension of the simple form:

$$\mathscr{R}_c(y_i, y_f, A_1, A_2, A_3, t_f) = y_i + (y_f - y_i)\frac{t}{t_f} + A_2 t(t - t_f) + A_3 t(t - t_f)\left(t + \frac{1}{2}t_f\right) + A_4 t(t - t_f)\left(t + \frac{2}{3}t_f\right)\left(t + \frac{1}{3}t_f\right) \tag{5}$$

where A_1, A_2 and A_3 correspond to the 3rd, 4th and 5th order polynomial terms respectively with each polynomial having evenly spaced roots between $t = 0$ and $t = t_f$. As with the simple parametrization t_f specifies the end of the ramps in time.

In each of the three ramps being optimized, the parameters y_i, y_f, A_1, A_2, A_3 are independent. However, the final time t_f is common.

Results of the optimization process

Figure 1 shows the optimal evaporation ramps for each of the five optimization runs discussed in the paper. It can be seen that the lower parameter searches (ML7p, ML6p and NM7p) converged to similar shaped ramps, whereas the higher dimensional searches found quite different optima. Table 1 outlines the optimal values for each parameter found in the 16 parameter MLOO run.

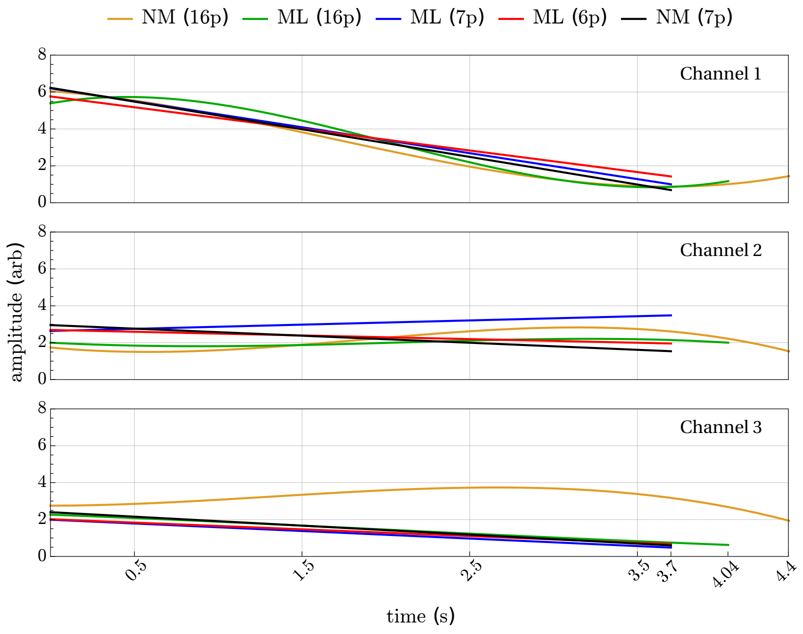

Figure 1. Optimal evaporation ramps for each of the five optimization runs presented in the main manuscript. The three separate plots represent each of the three experimental channels with each of the coloured lines corresponding to one of the five optimization runs. The low parameter searches (ML7p, ML6p and NM7p) consist of the simple parameterization which allows for linear evaporation ramps, whereas the higher parameter searches (ML16p, NM16p) use the complex parameterization allowing higher order terms to appear as well as allowing the total time of the evaporation ramps to be optimized.

	channel		
parameter	1	2	3
y_i	5.39066	2.	2.265
y_f	1.17105	2.	0.623434
A_1	-2.68112×10^{-6}	-0.0000924388	-0.000363626
A_2	0.317441	-0.0629076	0.00674956
A_3	-0.0020399	-0.00344333	0.
t_f	4.041276		

Table 1. Optimal parameter values for the 16 parameter machine learning optimization run.

References

1. Rasmussen, C. E. & Williams, C. K. I. *Gaussian Processes for Machine Learning* (The MIT Press, 2006).
